# Factors Influencing Posttraumatic Growth Among Childhood and Adolescent Cancer Survivors: A Scoping Review

**DOI:** 10.7759/cureus.100957

**Published:** 2026-01-06

**Authors:** Yuta Kogumazaka, Tae Kawahara, Akemi Yamazaki

**Affiliations:** 1 Division of Health Sciences, Graduate School of Medicine, The University of Osaka, Suita, JPN

**Keywords:** adolescent and young adults, childhood cancer survivors, pediatric psychology, posttraumatic growth, psycho-oncology

## Abstract

Cancer experiences are characterized by significant stress and life disruption. In childhood and adolescent cancer survivors, these experiences are often traumatic and accompanied by psychosocial burdens. However, increasing attention has recently been directed toward positive psychological changes following cancer, known as posttraumatic growth (PTG). Research on PTG in childhood and adolescent cancer survivors remains an emerging field. No study has comprehensively reviewed the factors influencing PTG in this population. In this scoping review, we systematically investigated theories and measures related to PTG among childhood and adolescent cancer survivors and evaluated factors influencing PTG in this group. This review followed the Joanna Briggs Institute methodology for scoping reviews and addressed the following questions: (1) What factors influence PTG levels among childhood and adolescent cancer survivors?, (2) Do these factors promote or inhibit PTG?, and (3) Which tools have been used to measure PTG among childhood and adolescent cancer survivors? The data sources included MEDLINE, Cumulative Index to Nursing and Allied Health Literature (CINAHL), PsycINFO, and ProQuest. Studies were mapped based on their characteristics, design, participant population, measurement tools, and key findings. A peer-reviewed search strategy developed by the university’s Life Sciences Librarian identified 1,715 studies, of which 44 met the eligibility criteria. The extracted factors were classified into six categories: cancer-related, personal, cognitive processing, social and interpersonal, coping, and psychological health and outcome-related factors. Qualitative research frequently identified social and interpersonal factors, suggesting an association between PTG and relationships with others. However, quantitative research primarily assessed the relationship between PTG and cancer-related as well as personal factors. Although social and interpersonal factors were also examined, the results varied depending on the PTG assessment scale. To advance our understanding of the subject, further research is warranted using the PTG Inventory, a PTG measurement tool validated across various fields.

## Introduction and background

Significant advances in medical treatment have markedly improved survival rates among children with cancer [1,2]. Nevertheless, childhood cancer remains a profoundly distressing life event that imposes substantial psychological and physical burdens on survivors and their families throughout diagnosis, treatment, and long-term follow-up [3]. Consequently, the psychosocial challenges faced by childhood and adolescent cancer survivors have been extensively examined [4]. However, more recently, attention has shifted toward the positive psychological changes, including thinking about life and treating others [5], that may emerge following traumatic cancer experiences. Notably, childhood and adolescent cancer survivors have been reported to demonstrate positive outcomes related to their illness, commonly referred to as posttraumatic growth (PTG) [5,6].

PTG is a concept introduced by Tedeschi and Calhoun to describe the positive psychological changes that can arise from struggling with major crises or traumatic life events [7]. They identified five core domains of PTG - relating to others, discovering new possibilities, personal strength, spiritual change, and appreciation of life [8]. The authors emphasize that PTG does not merely comprise recovery, but rather a trauma-triggered psychological transformation. According to the model of Calhoun and Tedeschi [9], trauma shatters the core beliefs of an individual, causing cognitive confusion and distress. In the early stages, individuals experience automatic, distressing thoughts (intrusive rumination). Over time, individuals consciously start trying meaning-making (deliberate rumination). Within relationships with others, self-disclosure through speaking or writing is encouraged, and this process facilitates growth.

Previous research on PTG among childhood and adolescent cancer survivors has revealed associations between PTG and both demographic and treatment-related characteristics [5,6], as well as its relationships with psychosocial functioning [10,11] and cognitive processes [12]. There is a growing need for strengthening the evidence base, not only for interventions that mitigate the negative effects of cancer survivorship but also for those that foster positive psychological outcomes. Recent reviews by Turner et al. [13] and Berkman et al. [14] have investigated PTG in childhood and adolescent cancer survivors; however, these were limited by their focus on quantitative studies or literature published between 2000 and 2018. Although research on PTG is expanding, whether the existing studies provide a sufficiently comprehensive understanding of the factors influencing PTG in this population remains unclear. Therefore, the present scoping review aimed to broadly map these influencing factors to enhance current evidence and identify knowledge gaps for future research.

## Review

Methods

Study Design

Scoping reviews are a form of evidence synthesis designed to map the existing literature on a specific topic and systematically identify research gaps [15]. The present review followed the methodological framework proposed by Arksey and O’Malley [16], further refined by the Joanna Briggs Institute (JBI) [17]. It was conducted in accordance with the Preferred Reporting Items for Systematic Reviews and Meta-Analyses (PRISMA)-ScR guidelines [15] and a modified six-stage framework outlined by Levac et al. [18].

Review Question

The aim of this review was to systematically evaluate the theories and measurement scales related to PTG among childhood and adolescent cancer survivors and to map the factors that influence PTG in this population. Guided by preliminary research, the scoping review addressed the following research questions:

1. What factors influence PTG among childhood and adolescent cancer survivors?

2. Do these factors promote or inhibit PTG?

3. What instruments have been used to measure PTG among childhood and adolescent cancer survivors?

Based on these questions, we reviewed the existing studies to determine whether relevant factors had been adequately investigated and to identify gaps in the evidence regarding their effects on PTG.

Eligibility Criteria

The inclusion criteria were as follows: (a) Population: patients diagnosed with any type and stage of cancer and aged 19 years or younger. (b) Concept: PTG. (c) Context: post-treatment. The exclusion criteria were a follows: studies in which the target population could not be separated into cancer and non-cancer patients, studies not written in English, and studies that did not examine or extract factors associated with PTG. Studies with populations comprising 50% or more participants aged 19 years or younger at the time of diagnosis were included. Studies were considered even if the exact age distribution of participants at diagnosis was not determined, as long as they indicated that the mean or median age of diagnosis was 19 years or younger. Concerning PTG measurement tools, we only included studies that explicitly mentioned the tool used in the given study for PTG measurement.

Peer-reviewed original articles employing all methodological approaches, including quantitative, qualitative, and mixed methods, were considered. Regarding other eligible articles (e.g., reviews, editorials, letters/communications), the cited references were screened; original articles matching the criteria were included in this review. Furthermore, the treatment of gray literature, such as conference proceedings and dissertations, was limited to materials published within the past three years (January 2023 or later) that were retrieved from the databases used for the search. Gray literature was also used for screening the cited references.

Search Strategy and Selection Process

We conducted a search on September 16, 2025, using the following online bibliographic databases: MEDLINE, Cumulative Index to Nursing and Allied Health Literature (CINAHL), PsycINFO, and ProQuest. The search strategy included terms related to the Population, Concept, and Context framework, such as cancer, child, and PTG, and was peer-reviewed by a life sciences librarian at our university. Our full search strategies are shown in the Appendix.

**Figure 1:**
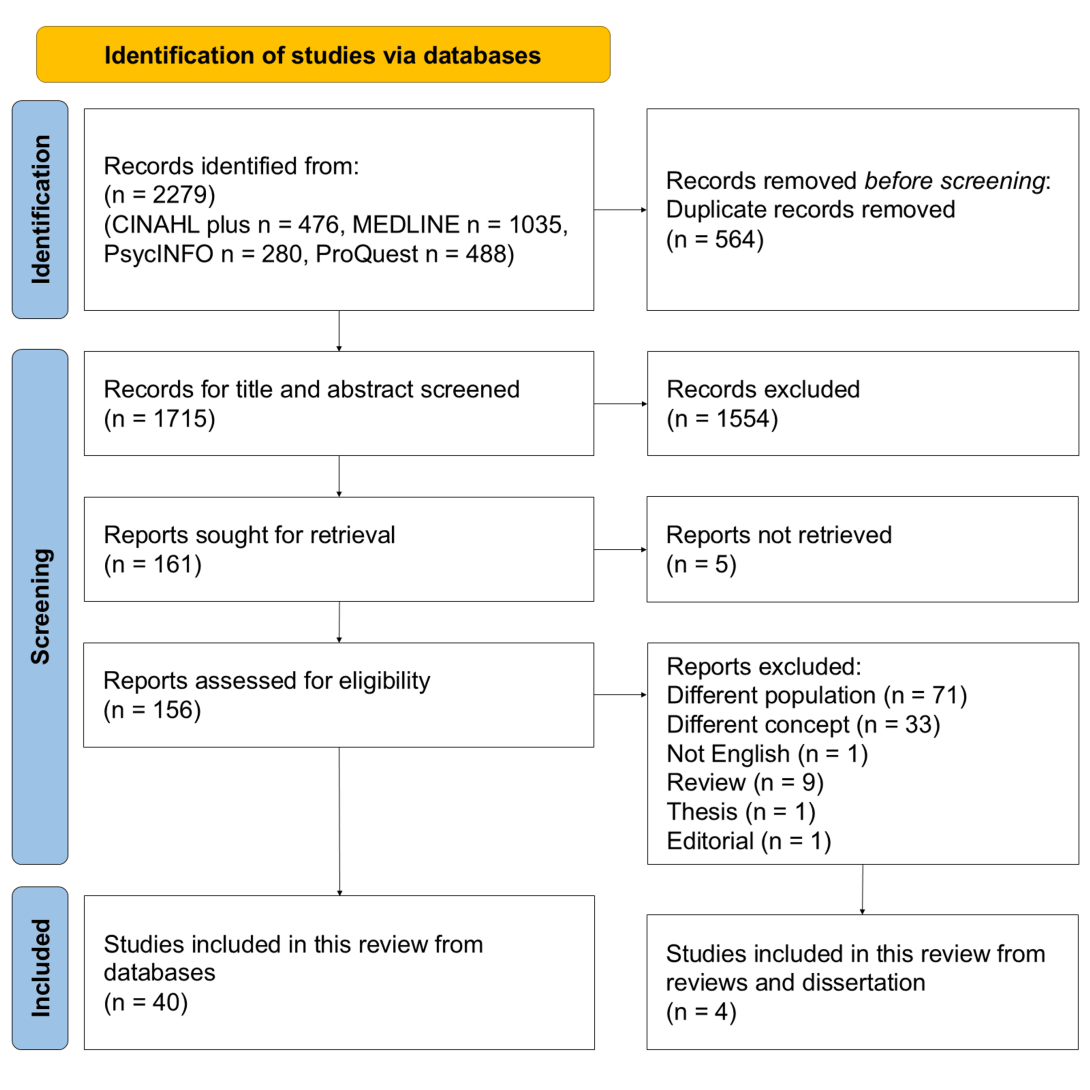
Preferred Reporting Items for Systematic Reviews and Meta-Analyses (PRISMA) Flowchart Depicting the Review Process

We screened eligible studies according to the Systematic Reviews and Meta-Analyses extension for scoping reviews guidelines established by Tricco et al. [15]. The online applications EndNote (Clarivate, London, UK) and Rayyan [19] were used to screen eligible studies. First, two reviewers (YK and AY) independently screened the titles and abstracts of all retrieved records using Rayyan to assess their eligibility based on the predefined inclusion criteria. As a result, there was a 6% disagreement in opinions. However, these disagreements were discussed by the two reviewers who conducted the screening. Considering any disagreements that remained unresolved, we had planned to request an opinion of a third reviewer (TK); however, no such studies were encountered. The study selection process is illustrated in the PRISMA flow diagram (Figure 1).

Data Collection

Two reviewers (YK and AY) created the data chart using Microsoft Excel (Microsoft Corporation, Redmond, WA, USA) after discussing the items to include. The first author extracted the study characteristics into the data chart, including the first author’s name, publication year, study country, design, participants and sample size, key findings, PTG measurement tools, and effects on PTG (promoting/inhibiting). The primary objective of this review was to comprehensively identify factors associated with PTG in childhood and adolescent cancer survivors. In this review, statistical significance of p <0.05 as indicated in the included studies was considered relevant. Factors associated with PTG were summarized descriptively in a table indicating the direction of association for each factor. Furthermore, those factors were summarized and named after rigorous discussion among all authors. Research gaps were identified and described based on the summarized results. Since the objective of this review was to map existing evidence, the risk of bias in individual studies was not assessed [15].

Ethical Consideration and Consultation

This scoping review did not require ethical considerations, as it involved the collection and summarization of data from previously published sources. According to Arksey and O'Malley [16] and Levac et al. [18], the consultation process can provide decisions and recommendations beyond the published research. This review did not include patients or the general population.

Results

Research Selection

After excluding duplicates, 1,715 studies were included for analysis. Of these, 1,554 were excluded after title and abstract screening. Of the 161 remaining studies, 156 were reviewed in full text (excluding five in which the full text was unavailable), and 40 met the inclusion criteria. Among these, nine review articles and one dissertation that satisfied the eligibility criteria were also included; after removing duplicates, four studies [20-23] remained. In total, 44 studies were incorporated into this review.

Characteristics of the Studies

Table 1 summarizes the 44 studies included in this review. Of these, 35 studies employed quantitative designs, seven used qualitative designs, and two adopted mixed-methods approaches. The studies were distributed across time periods as follows: two in the 1990s, two in the 2000s, 27 in the 2010s, and 13 in the 2020s. The most widely used PTG measurement tool was the Posttraumatic Growth Inventory (PTGI) developed by Tedeschi and Calhoun [8], utilized in 24 studies. Other instruments included the Benefit-Finding Scale for Children (BFSC) [24] and Benefit-Burden Scale for Children (BBSC) [25]. Based on Calhoun and Tedeschi’s PTG framework, we extracted factors related to PTG from the 44 studies and classified them into six categories of factors: cancer-related, personal, cognitive processing, social and interpersonal, coping, and psychological health and outcome-related factors.

**Table 1:** Summary of Study Characteristics CCS, childhood cancer children; AYA, adolescent and young adults; PTGI, Posttraumatic Growth Inventory; PTSS, posttraumatic stress symptoms; PPR, German translation of the PTGI; BBSC, Benefit-Burden Scale for Children; BFSC, Benefit-Finding Scale for Children; FCR, fear of cancer recurrence.

First Author (Year)	Country	Population	Design	Scale	Key Findings
Arpawong et al. (2013) [10]	USA	CCS diagnosed with cancer before the age of 18 (11-21 years), n=94	Quantitative	PTGI	PTG positively correlates with psychosocial functioning (p=0.006) and PTSS (p=0.02) and negatively correlates with physical functioning (p=0.04) and depression (p=0.0006).
Atay Turan et al. (2023) [26]	Turkey	AYA survivors (age at survey: 12-25 years), n=78	Quantitative	PTGI	Strong positive correlation between resilience and PTG (p<0.001). Resilience explains PTG to a certain extent.
Barakat et al. (2006) [5]	USA	Adolescent survivors (age at survey: 11-19 years), n=150; mothers, n=146; fathers, n=107	Quantitative	Perceptions of Changes in Self (PCS)	Subjective perceptions of treatment severity (p<0.01) and life-threateningness (p<0.005) were associated with PTG. PTG and PTSS exhibited a positive correlation (p<0.005).
Cantrell and Conte (2016) [27]	USA	CCS (age at survey: 19-22 years), n=5	Qualitative	Not applicable	The experience of receiving support from many people made CCS want to repay that kindness to others.
Cheng et al. (2016) [28]	Taiwan	Aboriginal adolescent survivors (age at diagnosis: 2-16 years, age at survey: 12-17 years), n=11	Qualitative	Not applicable	A qualitative study among aboriginal adolescent survivors identified “transformation and growth” as a recovery process.
Cook et al. (2021) [29]	USA	CCS (age at survey: 13-23 years), n=196	Quantitative	BBSC	The centrality of cancer events is associated with both PTG and PTSS (p<0.05). Centrality is higher in women.
de Castro et al. (2024) [30]	Brazil	CCS (age at diagnosis (mean): 10.24±5.15 years, age at survey (mean): 17.74±3.93 years), n=62	Quantitative	PTGI	Cluster analysis identified three distinct profiles: high PTG, high PTSS, and no change. PTG and PTSS were found to have independent structures, with rumination and challenge to core beliefs mediating their relationship.
Ekim and Ocakci (2015) [20]	Turkey	Adolescents with cancer (age at diagnosis: 9-16 years, age at survey: 12-18 years), n=108	Quantitative	PTGI	This study indicated that social support promotes a high PTG.
Ernst et al. (2023) [31]	Germany	CCS (age at diagnosis (mean): 6.3±4.4 years), n=633	Quantitative	PPR-5	Associations with personal characteristics, social factors, psychological factors, and coping-related factors were demonstrated.
Gianinazzi et al. (2016) [6]	Switzerland	CCS (age at diagnosis ≤ 16 years), n=309	Quantitative	PTGI	Uni- and multivariate analyses revealed the PTG-associated cancer-related characteristics. The effects of psychological distress, recurrence, and late effects became non-significant in the multivariate analysis.
Gunst et al. (2016) [32]	Germany	Adolescent cancer survivors (age at diagnosis: 15-18 years), n=784	Quantitative	PTGI	This study presented associations primarily with fear of death (p<0.001), psychosocial support (p<0.001), and symptoms of depression (p<0.05).
Howard Sharp et al. (2015) [21]	USA	CCS (age at survey: 8-19 years), n=153	Quantitative	BBSC	Human connections were strongly associated with PTG (p<0.039).
Howard Sharp et al. (2017) [22]	USA	Cancer survivors (age at survey: 8-21 years), n=201	Quantitative	BBSC	The study clarified differences in parental distress responses based on cancer experience versus non-experience, suggesting parental supportive responses as a primary factor directly related to PTG (p<0.001).
Karian et al. (1998) [33]	USA	CCS (age at diagnosis ≤ 18 years, age at survey: 23-26 years), n=5	Qualitative	Not applicable	A phenomenological study of childhood cancer experiences with findings supporting the theoretical framework of Health as Expanding Consciousness (Newman M.).
Kim (2017) [34]	Korea	CCS (age at survey: 15-28 years), n=15	Qualitative	Not applicable	Long-term survivors perceived self-directed life, normality in life, and inner maturity as positive growth, concretely illustrating the conceptual aspects of PTG (intrapersonal, social, and meaning-making growth).
Kim (2022) [11]	Korea	CCS who had been diagnosed with cancer during childhood and adolescence (age at survey: 11-30 years), n=117	Quantitative	PTGI (Korea ver.)	PTG was significantly associated with both hope (p<0.001) and self-efficacy (p=0.001). In the multivariate analysis, hope (p<0.001), the presence of siblings (p=0.038), and high economic status (p=0.009) were significant predictors of PTG.
Kim and Park (2019) [35]	Korea	Pediatric cancer survivors (age at survey: 11-13 years), n=6	Quantitative	PTGI (Korea ver.)	Implementation of exercise and play intervention in CCS resulted in significant increases in indicators including PTG (p=0.03), QOL (p=0.04), and physical strength levels (p=0.04).
Klosky et al. (2014) [36]	USA and Canada	CCS (age at diagnosis: 0-20 years), n=6,162	Quantitative	PTGI	After controlling for sociodemographic and medical variables, a weak positive correlation was shown between PTSS and PTG.
Koutná et al. (2017) [37]	Czechia	CCS diagnosed before the age of 18 (age at survey: 11-25 years), n=97	Quantitative	BFSC	Personal factors associated with PTG. Parental warmth strongly promoted PTG (p<0.001).
Koutná et al. (2022) [38]	Czechia	Child and adolescent survivors (age at diagnosis: 3.72±2.01 years (child), 8.18±3.93 years (adolescent), age at survey: 11-25 years), n=172	Quantitative	BFSC	After controlling for age, gender, and years since treatment, PTG remained positively associated with “outlook on life,” “social functioning,” and “intimate relationships” in the adolescent group (p<0.05). No significant association was detected in the children group.
Koutná et al. (2021) [39]	Czechia	CCS diagnosed before the age of 18 (age at survey: 11-27 years), n=167	Quantitative	BFSC	No significant difference was shown between PTG and PTSS, and FCR showed a weak correlation with PTG (p<0.05).
McDonnell et al. (2018) [40]	USA	CCS (age at diagnosis: 14-20 years), n=153	Quantitative	PTGI	PTG showed a slight decrease over time. The longitudinal analysis indicated that there was no significant difference between the effect of cancer-related worry on PTG and that of PTG on worry.
Molinaro and Fletcher (2018) [41]	Canada	Pediatric cancer survivors (age at diagnosis: 2-19 years), n=10	Qualitative	Not applicable	Cancer experience strengthened their “appreciation, relationships, and sense of purpose in life”.
Novakovic et al. (1996) [42]		CCS (age at treatment (mean): 15.8±5.3 years), n=85	Qualitative	Not applicable	This study yielded three themes: “negative experiences of cancer”, “positive experiences of cancer”, and “advice to newly diagnosed patients”.
Peloso et al. (2022) [12]		AYA survivors (age at diagnosis ≤ 18 years), n=43	Quantitative	PTGI	The Core Beliefs Challenge was the strongest predictor of PTG (p≤0.001). Rumination showed a positive correlation with PTG but did not emerge as an independent predictor in the multiple regression.
Rosales et al. (2021) [43]	USA	CCS (age at diagnosis: 5-18 years), n=116	Quantitative	The Child Health and Illness Profile–Adolescent Edition (CHIP-AE)	This study demonstrated correlations between PTG (i.e., sense of achievement and resilience) and ethnicity as well as educational background.
Sedmak et al. (2020) [44]	Croatia	AYA (age at diagnosis: 1-18 years, age at survey: 15-30 years), n=83	Quantitative	PTGI	Young people with more pronounced treatment consequences (i.e., high-level negative effects) reported significantly lower posttraumatic growth (p≤0.01).
Seitz et al. (2011) [45]	Germany	Adult long-term cancer survivors (age at diagnosis: 15-18 years, age at survey (mean): 30.44±6.05 years), n=820	Quantitative	PTGI (Germany ver.)	PTG displayed a positive correlation with life satisfaction in adolescent patients with cancer (p<0.001).
Slaughter et al. (2020) [46]	USA	CCS (age at survey: 15-25 years), n=129	Quantitative	PTGI-SF	Low-level parental negative mental health was associated with positive mental health in children.
Slaughter et al. (2022) [47]	USA	Hispanic CCS (age at diagnosis ≤ 18 years, age at survey (mean): 19.35±2.77 years), n=68	Quantitative	PTGI-SF	Acculturation discrepancy between parents and children affects PTG and psychological QOL. Specifically, when children are more acculturated to American culture, PTG and QOL tend to be higher (p<0.01).
Tobin et al. (2018) [48]	USA	CCS (age at diagnosis: 5-19 years, age at survey: 15-25 years), n=235	Quantitative	PTGI-SF	In any culture, a strong cultural identity may promote PTG after cancer.
Tremolada et al. (2016) [49]	Italy	CCS (age at diagnosis <18 years, age at survey: 15-25 years), n=223	Quantitative	The Personal Growth Inventory	Personal growth was most significantly influenced by personal and cancer-related factors.
Tremolada et al. (2018) [50]	Italy	CCS (age at diagnosis < 18 years, age at survey: 15-25 years), n=100	Mixed	Ecocultural Family Interview-Cancer (EFI-C)	The potential for positive relationships with healthcare professionals and family members, along with the treatment memories and narrative skills, to promote personal growth (p<0.05).
Turner-Sack et al. (2013) [51]	Canada	Adolescents with cancer (age at diagnosis: 2-17 years), n=31	Quantitative	PTGI	This study demonstrated that the prediction of high risk of recurrence (p<0.001) and acceptance coping strategies (p<0.01) enhance PTG.
Weinstein et al. (2018) [52]	USA and Canada	CCS (age at diagnosis ≤ 10 years, baseline 12-17 years), n=2802	Quantitative	PTGI	Predictors of higher PTG in adolescents included older age at diagnosis (p=0.001), experiencing more severe chronic health conditions (p=0.01), cancer recurrence/relapse (p=0.01), and being diagnosed with a non-CNS cancer (p=0.02).
Wicks and Mitchell (2010) [23]	New Zealand	Cancer survivors (age at diagnosis: 14-19 years), n=10	Qualitative	Not applicable	A qualitative study of “adolescent cancer experiences” identified that participants recognized benefits from their cancer experience, including improved personal attributes, strengthened relationships, and material gains.
Wilson et al. (2016) [53]	USA	Children and adolescents (age at survey: 7-18 years), n=61	Quantitative	BBSC	In both univariate and multivariate analyses, PTSS (p<0.01) and stronger parent positive relations (p<0.05) and positive family functioning (p<0.05) were associated with higher levels of PTG.
Wurz et al. (2022) [54]	Canada	CCS (age at diagnosis < 18 years, age at survey: 9-25 years), n=113	Quantitative	BBSC	In the univariate analysis, no association was detected between PTSS and PTG. In the multivariate analysis, parental PTSS did not influence PTG in children.
Yi and Kim (2014) [55]	Korea	Korean cancer survivors (age at diagnosis < 19 years, age at survey: 15-39 years), n=225	Quantitative	PTGI (Korea ver.)	No curvilinear relationship was demonstrated between PTSS and PTG; instead, an inverse correlation was shown.
Yi et al. (2015) [56]	USA	CCS (age at diagnosis: 0-21 years, age at survey: 18-39 years), n=602	Quantitative	PTGI	The primary determinants of PTG were established as follows: being female (p<0.05), non-white (p<0.05), and older adult at diagnosis (p<0.05) as well as displaying non-solid tumors (p<0.05), high optimism (p<0.01), and high social support (p<0.01).
Yi and Nam (2017) [57]	Korea	CCS (age at diagnosis: 2-18 years, age at survey: 15-39 years), quantitative n=145; qualitative n=30	Mixed	PTGI	Qualitative research identified participation in advocacy and disclosure of medical history as coping strategies for stigma, suggesting they may be elements of growth. Quantitative research showed associations in some subscales of PTG (p<0.05).
Yonemoto et al. (2009) [58]	Japan	Long-term survivors of osteosarcoma (age at diagnosis<20 years), n=30	Quantitative	PTGI (Japan ver.)	Factors promoting PTG included social support (p=0.0002), older age at diagnosis (p=0.0197), and amputation experience (p=0.0031), while good family functioning was identified as a factor suppressing PTSS.
Yuen et al. (2014) [59]	China	CCS (age at diagnosis < 17 years, age at survey: 17.2-31.3 years), n=89	Quantitative	PTGI (China ver.)	Hope was shown to be a significant positive factor in PTG among CCS (p<0.01). Furthermore, it was suggested that positive rumination may mediate the relationship between hope and PTG.
Zebrack et al. (2012) [60]	USA and Canada	CCS (age at diagnosis ≤ 20 years, age at survey ≥ 18 years), n=6,425	Quantitative	PTGI	Perceived positive impact is associated with female gender (p<0.01), older age at diagnosis (p<0.01), relapse or second malignancy (p<0.01), and intensity of treatment (p<0.01).

Trends in Factors Related to PTG in Quantitative Research

Table 2 and Table 3 summarize the factors influencing PTG identified from quantitative studies. Numerous studies assessed cancer-related and personal factors. Among the cancer-related factors, age at diagnosis, recurrence or secondary cancer, older age at diagnosis, and the experience of repeated treatment were frequently associated with higher PTG. Regarding personal factors, female patients generally reported higher PTG than male patients, although a few studies showed no significant gender differences. In terms of psychological health and outcome-related factors, the relationship between posttraumatic stress disorder (PTSD) or posttraumatic stress symptoms (PTSS) and PTG has been a major focus of investigation.

**Table 2:** Summary of Univariate Analysis Findings +, Positive correlation; -, Negative correlation; *^1^, Differences in PTG levels were observed across cancer types, with lower levels tending to be seen in central nervous system tumors and some solid tumors compared to leukemia; *^2^, Hispanics tend to show lower PTG than non-Hispanics. However, the opposite result was also shown. PTSD, posttraumatic stress disorder; PTSS, posttraumatic stress symptoms; QoL, quality of life; PTG, posttraumatic growth.

Factors		Direction of the Relationship
Cancer-related factors	Age at diagnosis	+ [5,6,20,31,37,50,56]
	Treatment intensity	+ [5]
	Treatment duration	+ [6,20]
	Recurrence/secondary cancer	+ [6]
	Cancer type	*^1^ [10,52,56]
	Duration since diagnosis	- [5]
	Treatment-related comorbidity/late effects	+ [32]
Personal factors	Gender (ref=male)	+ [20,32,50], - [10,56]
	Age at study	+ [20,21,31,50]
	Optimism	+ [10,56]
	Educational level	+ [43,50]
	Economic status	+ [11]
	Marital status	+ [31]
	Ethnicity	*^2^ [10,43,46,47]
	Siblings (yes/no)	+ [11]
Cognitive processing factors	Rumination/self-reflection	+ [12,30,59]
	Challenge to core beliefs	+ [12,30]
	Cancer experience centrality	+ [22,29]
	Predictions of remaining in remission	- [51]
Social and interpersonal factors	Family support and warmth	+ [11,22]
	Family (parents, siblings) relationships	+ [21,50,53]
	Family function	+ [53]
	Support from healthcare professionals	+ [50,53]
	Social support	+ [20,56,58]
	Participation in advocacy	+ [57]
Coping factors	Religious worship and belief	+ [53]
Psychological health and outcome-related factors	Depression	- [10,32,37]
	PTSD/PTSS	+ [5,21,30,36,49,53], - [10,31]
	Parent’s PTSD	+ [46]
	Resilience	+ [26,31]
	QoL	+ [10,38], - [38]
	Hope	+ [11,59]
	Fear of cancer relapse	+ [39]

**Table 3:** Summary of Multivariate Analysis Findings +, Positive correlation; -, Negative correlation; *^1^, Differences in PTG levels were observed across cancer types, with lower levels tending to be seen in central nervous system tumors and some solid tumors compared to leukemia; *^2^, Non-white adults showed higher PTG, but Hispanic and non-Hispanic groups demonstrated different directions of relationship; Curvilinear, Religious service attendance had an inverted U relationship with PTG, with the highest PTG scores at moderate levels. That is, the highest levels/lowest levels of religious service attendance decrease PTG. PTSD, posttraumatic stress disorder; PTSS, posttraumatic stress symptoms; PSS, Parental Stress Scale; QoL, quality of life; PTG, posttraumatic growth.

Factors		Direction of the Association
Cancer-related factors	Age at diagnosis	+ [6,36,52,56,58,60], - [40,49]
	Treatment intensity	+ [60]
	Treatment duration	+ [6]
	Recurrence/secondary cancer	+ [6,36,52,60]
	Cancer type	*^1^ [10,56,60]
	Duration since diagnosis	- [60]
Personal factors	Gender (ref=male)	+ [6,36,37,49,56,60]
	Age at study	+ [37,49,55]
	Optimism	+ [56]
	Educational level	+ [60]
	Economic status	+ [11]
	Marital status	+ [60]
	Ethnicity	*^2^ [10,36,48]
	Siblings (yes/no)	+ [11]
Cognitive processing factors	Rumination/self-reflection	+ [59]
	Challenge to core beliefs	+ [12]
	Cancer experience centrality	+ [22]
	Predictions of remaining in remission	- [51]
Social and interpersonal factors	Family support and warmth	+ [22,37]
	Family (parents, siblings) relationships	+ [21]
	Support from healthcare professionals	+ [50]
	Social support	+ [56,58]
Coping factors	Religious worship and belief	Curvilinear [48]
	Coping strategy	+ [51]
Psychological health and outcome-related factors	Depression	- [10,32]
	PTSD/PTSS	+ [5,10,36,53]
	Parent’s PSS	- [46]
	QoL	+ [10,38,44,45]
	Hope	+ [11,59]

Trends in Factors Related to PTG in Qualitative Research

Table 4 summarizes the factors related to PTG identified from qualitative studies. Social and interpersonal factors received most of the support across the studies. A distinctive result in the qualitative studies was the frequent extraction of narrativization and meaning-making of experience under cognitive processing factors. Sharing cancer experiences with others enabled meaning-making and growth.

**Table 4:** Summary of Qualitative Research Findings +, Positive correlation.

Factors		Relationship
Cancer-related factors	Experience of fighting illness	+ [23,27,33]
Personal factors	Autonomy	+ [34,57]
	Optimism	+ [33]
Cognitive processing factors	Narrativization and meaning-making of experience	+ [34,42,57]
	Rumination/self-reflection	+ [28,34]
Social and interpersonal factors	Cooperation and support from family, peers, and healthcare professionals	+ [23,27,28,33,34,41,42]
Coping factors	Religious and cultural beliefs	+ [28]
Psychological health and outcome-related factors	Changing value and outlook on life	+ [27,28,33,34]

Discussion

This review represents the first comprehensive mapping of factors associated with PTG among childhood and adolescent cancer survivors. The findings indicated that research exploring the relationship between PTG and childhood and adolescent cancer survivors has increased in recent years and is gaining substantial scholarly attention. The 44 reviewed studies examined a wide range of factors related to PTG, including cancer-related, personal, cognitive processing, social and interpersonal, coping, and psychological health and outcome-related factors. Although the PTGI [8] was the most commonly used measurement tool, several studies employed the BFSC [24] or BBSC [25]. These scales assess benefit-finding (BF), a concept closely related to but distinct from PTG [61-63]. Previous studies have compared BF and PTG, generally acknowledging their conceptual overlap while emphasizing their theoretical distinction, except Applebaum et al. [61]. This review identified a notable difference between the measures: while studies using the PTGI frequently reported associations with cancer-related and personal factors, those using the BBSC or BBFC showed little evidence of such associations. Furthermore, although some studies employing alternate measures reported results similar to those obtained with the PTGI, it remains possible that, similar to BF, these measures capture different dimensions of positive adaptation.

Qualitative and mixed-methods studies consistently reported positive psychological effects associated with the experience of fighting cancer, cancer-related factors [23,27,33]. These findings underscore that cancer treatment, while profoundly stressful, can also serve as a catalyst for PTG among childhood and adolescent cancer survivors. A characteristic feature of qualitative studies was the emphasis on personal narratives and meaning in PTG development. This is classified as a cognitive processing factor in this study and aligns with the concept of “self-disclosure” described in Calhoun and Tedeschi’s [9] theoretical model of PTG. This further supports their proposed framework.

Focusing on the quantitative and mixed-methods studies, many did not perform multivariate analysis or did not report (NR) the results, highlighting the need for more reliable evidence adjusted for confounding factors, and notable research gaps remain. Cognitive processing and social or interpersonal factors have consistently shown positive associations with PTG. In contrast, relatively more studies have examined cancer-related and personal factors, but their findings have been inconsistent. For instance, among cancer-related variables, results concerning cancer type varied widely. Regarding personal factors, female sex was frequently associated with higher PTG, and older age at the time of assessment tended to correlate with higher PTG. However, several studies found no significant associations or reported conflicting outcomes, making drawing firm conclusions about these relationships difficult. These inconsistencies may reflect the influence of other, more dominant factors related to PTG development.

According to Calhoun and Tedeschi's [9] theoretical model of PTG, sociocultural influences are considered to play a significant role in PTG development and are predicted to have a substantial impact. Regarding that the PTGI [8] has been translated into several languages, validated, and widely used, variations in its factor structure have been reported [64,65], likely reflecting cultural aspects unique to different populations. Recent findings in German-speaking patients similarly revealed discrepancies in the PTGI’s factor structure, further supporting the role of cultural variability [66]. This review also identified several studies examining the relationship between ethnicity and PTG, but their results were inconsistent, possibly due to differences in the ethnic compositions and cultural contexts of the study samples. Further research is therefore needed to establish stronger evidence regarding the influence of ethnicity and culture on PTG among childhood and adolescent cancer survivors.

Thus, a clear gap between qualitative and quantitative studies has become evident. In qualitative research, social and interpersonal factors such as support from healthcare professionals and family members were emphasized in seven studies [23,27,28,33,34,41,42]. Quantitative studies have also consistently demonstrated positive associations between these factors and PTG; however, many of these studies [21,22,37,50,53] employed measurement tools other than the PTGI. Future research should conduct multivariate analyses incorporating social and interpersonal factors using the PTGI, the most widely used measure of PTG, to strengthen evidence in childhood and adolescent cancer survivors.

## Conclusions

In this review, we mapped six factors influencing PTG among childhood and adolescent cancer survivors and identified key research gaps. The methodology employed in this review does not allow for drawing conclusions on the strength of the association; it should be interpreted as indicating the existence of an association. Although quantitative studies have primarily assessed cancer-related and personal factors extensively, the other four factors have received relatively little attention. In contrast, qualitative research frequently identified social and interpersonal factors, suggesting an association between PTG and relationships with others. Although examined in quantitative studies, the results appeared to vary depending on the measurement scale used to assess PTG. Despite cultural and methodological challenges, further research applying the PTGI, a widely validated tool applicable beyond oncology contexts, would be essential to advance our understanding of PTG and strengthen the evidence base in childhood and adolescent cancer survivors.

## Appendices

**Table 5:** MEDLINE Search Strategy

No.	Formula	Result
1	exp Neoplasms/and (exp Child/or exp adolescent/or exp infant/)	445,080
2	((cancer or tumor or neoplasms or leukemia or lymphoma or neuroblastoma or retinoblastoma or histiocytosis or carcinoma or sarcoma) adj3 (child* or adolescent* or youth* or teen* or young* or son* or daughter* or girl* or boy* or AYA*)).ti,ab.	69,629
3	or/1-2	47,511
4	exp Survivorship/or exp Survivors/or exp Aftercare/	294,697
5	(Post treat* or Survivor* or (treat* adj3 complet*) or aftercare*).af.	294,012
6	or/4-5	519,275
7	and/3,6	20,605
8	exp "Adaptation, Psychological"/ or (posttraumatic growth or post-traumatic growth or stress-related growth or positive growth or adversarial growth or growth following adversity or benefit finding or perceived benefit or positive reframing or psychological adaptation).ti,ab.	152,693
9	and/7-8	1,062
10	limit 9 to English language	1,035

**Table 6:** CINAHL Search Strategy

No.	Formula	Result
1	MH "Neoplasms+" AND (MH "Child+" OR MH "Minors (Legal)" OR MH "Adolescence+")	73,148
2	TI (cancer or tumor or neoplasms or leukemia or lymphoma or neuroblastoma or retinoblastoma or histiocytosis or carcinoma or sarcoma) OR AB (cancer or tumor or neoplasms or leukemia or lymphoma or neuroblastoma or retinoblastoma or histiocytosis or carcinoma or sarcoma)	776,330
3	TI (child* or adolescent* or youth* or teen* or young* or son* or daughter* or girl* or boy* or AYA*) OR AB (child* or adolescent* or youth* or teen* or young* or son* or daughter* or girl* or boy* or AYA*)	1,045,318
4	S2 AND S3	69,605
5	S1 OR S4	117,875
6	MH "Survivorship+" or MH "Survivors+" or MH "Aftercare+"	35,868
7	TI (Post treat* or Survivor* or (treat* adj complet*) or aftercare*) OR AB (Post treat* or Survivor* or (treat* N3 complet*) or aftercare*)	94,503
8	S6 OR S7	105,933
9	S5 AND S8	10,303
10	MH "Adaptation, Psychological+" OR TI (posttraumatic growth or post-traumatic growth or stress-related growth or positive growth or adversarial growth or growth following adversity or benefit finding or perceived benefit or positive reframing or psychological adaptation) OR AB (posttraumatic growth or post-traumatic growth or stress-related growth or positive growth or adversarial growth or growth following adversity or benefit finding or perceived benefit or positive reframing or psychological adaptation)	58,433
11	S9 AND S10	480
12	S11; filter English	476

**Table 7:** PsycINFO Search Strategy

No.	Formula	Result
1	(DE "Neoplasms" OR DE "Benign Neoplasms" OR DE "Breast Neoplasms" OR DE "Childhood Neoplasms" OR DE "Digestive System Neoplasms" OR DE "Endocrine Neoplasms" OR DE "Leukemias" OR DE "Lung Neoplasms" OR DE "Metastasis" OR DE "Nervous System Neoplasms" OR DE "Skin Neoplasms" OR DE "Terminal Cancer") OR TI (cancer or tumor or neoplasms or leukemia or lymphoma or neuroblastoma or retinoblastoma or histiocytosis or carcinoma or sarcoma) OR AB (cancer or tumor or neoplasms or leukemia or lymphoma or neuroblastoma or retinoblastoma or histiocytosis or carcinoma or sarcoma)	103,512
2	(DE "Child+" OR DE "Minors (Legal)" OR DE "Adolescence+") OR TI (child* or adolescen* or youth* or teen* or young* or son* or daughter* or girl* or boy* or AYA*) OR AB (child* or adolescen* or youth* or teen* or young* or son* or daughter* or girl* or boy* or AYA*)	1,305,160
3	(DE "Survivorship+" or DE "Survivors+" or DE "Aftercare+") OR TI (Post treat* or Survivor* or (treat* NEAR/3 complet*) or aftercare*) OR AB (Post treat* or Survivor* or (treat* adj complet*) or aftercare*)	57,888
4	(DE "Adaptation" OR DE "Environmental Adaptation" OR DE "Sensory Adaptation" OR DE "Thermal Acclimatization" OR DE "Adversity" OR DE "Posttraumatic Stress" OR DE "Resilience (Psychological)" OR DE "Posttraumatic Growth") OR (posttraumatic growth or post-traumatic growth or stress-related growth or positive growth or adversarial growth or growth following adversity or benefit finding or perceived benefit or positive reframing or psychological adaptation) OR AB (posttraumatic growth or post-traumatic growth or stress-related growth or positive growth or adversarial growth or growth following adversity or benefit finding or perceived benefit or positive reframing or psychological adaptation)	92,241
5	S1 AND S2 AND S3 AND S4	289
6	S5; filter English	280

**Table 8:** ProQuest Search Strategy

No.	Formula	Result
1	TI,AB((cancer OR tumor OR neoplasm* OR leukemia OR lymphoma OR neuroblastoma OR retinoblastoma OR histiocytosis OR carcinoma OR sarcoma) NEAR/3 (child* OR adolescen* OR youth* OR teen* OR young* OR son* OR daughter* OR girl* OR boy* OR AYA*))	226,833
2	TI,AB((post treat* OR survivor* OR "treat* NEAR/3 complet*" OR aftercare*))	1,382,466
3	"posttraumatic growth" OR "post-traumatic growth" OR "stress-related growth" OR "positive growth" OR "adversarial growth" OR "growth following adversity" OR "benefit finding" OR "perceived benefit" OR "positive reframing" OR "psychological adaptation"	385,644
4	S1 AND S2 AND S3	641
5	S4;Filter;NOT MEDLINE	518
6	S5;Filter;English	488

## References

[1] Erdmann F, Frederiksen LE, Bonaventure A, Mader L, Hasle H, Robison LL, Winther JF (2021). Childhood cancer: Survival, treatment modalities, late effects and improvements over time. Cancer Epidemiol.

[2] Nakata K, Ito Y, Magadi W (2018). Childhood cancer incidence and survival in Japan and England: A population-based study (1993-2010). Cancer Sci.

[3] Sharp KM, Lindwall JJ, Willard VW, Long AM, Martin-Elbahesh KM, Phipps S (2017). Cancer as a stressful life event: Perceptions of children with cancer and their peers. Cancer.

[4] Kwak M, Zebrack BJ, Meeske KA (2013). Prevalence and predictors of post-traumatic stress symptoms in adolescent and young adult cancer survivors: A 1-year follow-up study. Psychooncology.

[5] Barakat LP, Alderfer MA, Kazak AE (2006). Posttraumatic growth in adolescent survivors of cancer and their mothers and fathers. J Pediatr Psychol.

[6] Gianinazzi ME, Rueegg CS, Vetsch J, Lüer S, Kuehni CE, Michel G (2016). Cancer's positive flip side: Posttraumatic growth after childhood cancer. Support Care Cancer.

[7] Calhoun LG, Tedeschi RG (1999). Facilitating Posttraumatic Growth: A Clinician's Guide.

[8] Tedeschi RG, Calhoun LG (1996). The Posttraumatic Growth Inventory: Measuring the positive legacy of trauma. J Trauma Stress.

[9] Calhoun LG, Tedeschi RG (2006). Handbook of Posttraumatic Growth: Research & Practice, 1st ed.

[10] Arpawong TE, Oland A, Milam JE, Ruccione K, Meeske KA (2013). Post-traumatic growth among an ethnically diverse sample of adolescent and young adult cancer survivors. Psychooncology.

[11] Kim Y (2022). Factors associated with post-traumatic growth in Korean survivors of childhood cancer. Oncol Nurs Forum.

[12] Peloso FC, Gonçalves T, Armiliato MJ, Gregianin L, Ramos C, de Castro EK (2022). Posttraumatic growth among childhood cancer survivors and their caregivers: Associations with rumination and beliefs challenge. Psicooncología.

[13] Turner JK, Hutchinson A, Wilson C (2018). Correlates of post-traumatic growth following childhood and adolescent cancer: A systematic review and meta-analysis. Psychooncology.

[14] Berkman AM, Robert RS, Roth M, Askins MA (2022). A review of psychological symptoms and post-traumatic growth among adolescent and young adult survivors of childhood cancer. J Health Psychol.

[15] Tricco AC, Lillie E, Zarin W (2018). PRISMA Extension for Scoping Reviews (PRISMA-ScR): Checklist and explanation. Ann Intern Med.

[16] Arksey H, O'Malley L (2005). Scoping studies: Towards a methodological framework. Int J Soc Res Methodol.

[17] Peters MD, Godfrey CM, Khalil H, McInerney P, Parker D, Soares CB (2015). Guidance for conducting systematic scoping reviews. Int J Evid Based Healthc.

[18] Levac D, Colquhoun H, O'Brien KK (2010). Scoping studies: Advancing the methodology. Implement Sci.

[19] Ouzzani M, Hammady H, Fedorowicz Z, Elmagarmid A (2016). Rayyan-a web and mobile app for systematic reviews. Syst Rev.

[20] Ekim A, Ocakci AF (2015). Relationship between posttraumatic growth and perceived social support for adolescents with cancer. J Hosp Palliat Nurs.

[21] Howard Sharp KM, Willard VW, Okado Y, Tillery R, Barnes S, Long A, Phipps S (2015). Profiles of connectedness: Processes of resilience and growth in children with cancer. J Pediatr Psychol.

[22] Howard Sharp KM, Willard VW, Barnes S, Tillery R, Long A, Phipps S (2017). Emotion socialization in the context of childhood cancer: Perceptions of parental support promotes posttraumatic growth. J Pediatr Psychol.

[23] Wicks L, Mitchell A (2010). The adolescent cancer experience: Loss of control and benefit finding. Eur J Cancer Care (Engl).

[24] Phipps S, Long AM, Ogden J (2007). Benefit Finding Scale for Children: Preliminary findings from a childhood cancer population. J Pediatr Psychol.

[25] Currier JM, Hermes S, Phipps S (2009). Brief report: Children's response to serious illness: Perceptions of benefit and burden in a pediatric cancer population. J Pediatr Psychol.

[26] Atay Turan S, Sarvan S, Akcan A, Guler E, Say B (2023). Adolescent and young adult survivors of cancer: Relationship between resilience and post-traumatic growth. Curr Psychol.

[27] Cantrell M, Conte TM (2016). From chemo to college: The college experience of childhood cancer survivors. J Pediatr Oncol Nurs.

[28] Cheng YC, Huang CY, Wu WW, Chang SC, Lee-Hsieh J, Liang SY, Cheng SF (2016). The lived experiences of aboriginal adolescent survivors of childhood cancer during the recovering process in Taiwan: A descriptive qualitative research. Eur J Oncol Nurs.

[29] Cook JL, Russell K, Long A, Phipps S (2021). Centrality of the childhood cancer experience and its relation to post-traumatic stress and growth. Psychooncology.

[30] de Castro EK, da Silva Oliveira JA, Armiliato MJ, Peloso F, Valentini F (2024). Profiles of posttraumatic growth and posttraumatic stress symptoms in childhood cancer survivors. J Child Adolesc Trauma.

[31] Ernst M, Werner AM, Brähler E, Wild PS, Faber J, Merzenich H, Beutel ME (2023). Posttraumatic growth after childhood cancer: Psychometric evaluation of a five-item short form and associations with mental health. J Psychosom Res.

[32] Gunst DC, Kaatsch P, Goldbeck L (2016). Seeing the good in the bad: Which factors are associated with posttraumatic growth in long-term survivors of adolescent cancer?. Support Care Cancer.

[33] Karian VE, Jankowski SM, Beal JA (1998). Exploring the lived-experience of childhood cancer survivors. J Pediatr Oncol Nurs.

[34] Kim Y (2017). Exploration of life experiences of positive growth in long-term childhood cancer survivors. Eur J Oncol Nurs.

[35] Kim Y, Park S (2019). Feasibility and benefits of a combined programme of exercise and play for paediatric cancer survivors: A pilot study. Eur J Cancer Care (Engl).

[36] Klosky JL, Krull KR, Kawashima T (2014). Relations between posttraumatic stress and posttraumatic growth in long-term survivors of childhood cancer: A report from the Childhood Cancer Survivor Study. Health Psychol.

[37] Koutná V, Jelínek M, Blatný M, Kepák T (2017). Predictors of posttraumatic stress and posttraumatic growth in childhood cancer survivors. Cancers (Basel).

[38] Koutná V, Blatný M, Jelínek M (2022). Posttraumatic stress and growth in adolescent childhood cancer survivors: Links to quality of life. Front Psychol.

[39] Koutná V, Blatný M, Jelínek M (2021). Posttraumatic stress and growth in childhood cancer survivors: Considering the pathways for relationship. J Psychosoc Oncol.

[40] McDonnell GA, Pope AW, Schuler TA, Ford JS (2018). The relationship between cancer-related worry and posttraumatic growth in adolescent and young adult cancer survivors. Psychooncology.

[41] Molinaro ML, Fletcher PC (2018). Taking lemons and making lemonade: Posttraumatic growth from pediatric cancer. Clin Nurse Spec.

[42] Novakovic B, Fears TR, Wexler LH, McClure LL, Wilson DL, McCalla JL, Tucker MA (1996). Experiences of cancer in children and adolescents. Cancer Nurs.

[43] Rosales P, Evangelista L, Guo Y, Agbayani CG, Kain ZN, Fortier MA (2021). Exploring differences in perceived satisfaction, resilience, and achievement between Hispanic and non-Hispanic White childhood cancer survivors. J Pediatr Health Care.

[44] Sedmak M, Bogdanić A, Grubić M (2020). Correlates of quality of life in pediatric cancer survivors. Psychiatr Danub.

[45] Seitz DC, Hagmann D, Besier T (2011). Life satisfaction in adult survivors of cancer during adolescence: What contributes to the latter satisfaction with life?. Qual Life Res.

[46] Slaughter RI, Hamilton AS, Cederbaum JA, Unger JB, Baezconde-Garbanati L, Milam JE (2020). Relationships between parent and adolescent/young adult mental health among Hispanic and non-Hispanic childhood cancer survivors. J Psychosoc Oncol.

[47] Slaughter RI, Hamilton AS, Cederbaum JA, Unger JB, Baezconde-Garbanati L, Milam JE (2022). Acculturation discrepancy and mental health associations among Hispanic childhood cancer survivors and their parents. Psychooncology.

[48] Tobin J, Allem JP, Slaughter R, Unger JB, Hamilton AS, Milam JE (2018). Posttraumatic growth among childhood cancer survivors: Associations with ethnicity, acculturation, and religious service attendance. J Psychosoc Oncol.

[49] Tremolada M, Bonichini S, Basso G, Pillon M (2016). Post-traumatic stress symptoms and post-traumatic growth in 223 childhood cancer survivors: Predictive risk factors. Front Psychol.

[50] Tremolada M, Bonichini S, Basso G, Pillon M (2018). Adolescent and young adult cancer survivors narrate their stories: Predictive model of their personal growth and their follow-up acceptance. Eur J Oncol Nurs.

[51] Turner-Sack AM, Menna R, Setchell SR, Maan C, Cataudella D (2013). "Posttraumatic growth, coping strategies, and psychological distress in adolescent survivors of cancer": Corrigendum. J Pediatr Oncol Nurs.

[52] Weinstein AG, Henrich CC, Armstrong GT, Stratton KL, King TZ, Leisenring WM, Krull KR (2018). Roles of positive psychological outcomes in future health perception and mental health problems: A report from the Childhood Cancer Survivor Study. Psychooncology.

[53] Wilson JZ, Marin D, Maxwell K (2016). Association of posttraumatic growth and illness-related burden with psychosocial factors of patient, family, and provider in pediatric cancer survivors. J Trauma Stress.

[54] Wurz A, Patton M, Merz EL, Hou SH, Cho S, Schulte F (2022). Exploring posttraumatic stress symptoms and posttraumatic growth among children living beyond cancer and their parents using an actor-partner interdependence model. Cancers (Basel).

[55] Yi J, Kim MA (2014). Postcancer experiences of childhood cancer survivors: How is posttraumatic stress related to posttraumatic growth?. J Trauma Stress.

[56] Yi J, Zebrack B, Kim MA, Cousino M (2015). Posttraumatic growth outcomes and their correlates among young adult survivors of childhood cancer. J Pediatr Psychol.

[57] Yi HJ, Nam SI (2017). The effect of advocacy for overcoming stigma on posttraumatic growth: Focusing on childhood cancer survivors. Soc Work Health Care.

[58] Yonemoto T, Kamibeppu K, Ishii T, Iwata S, Hagiwara Y, Tatezaki S-I (2009). Psychosocial outcomes in long-term survivors of high-grade osteosarcoma: A Japanese single-center experience. Anticancer Res.

[59] Yuen AN, Ho SM, Chan CK (2014). The mediating roles of cancer-related rumination in the relationship between dispositional hope and psychological outcomes among childhood cancer survivors. Psychooncology.

[60] Zebrack BJ, Stuber ML, Meeske KA (2012). Perceived positive impact of cancer among long-term survivors of childhood cancer: A report from the childhood cancer survivor study. Psychooncology.

[61] Applebaum AJ, Marziliano A, Schofield E, Breitbart W, Rosenfeld B (2021). Measuring positive psychosocial sequelae in patients with advanced cancer. Psychol Trauma.

[62] Harding S, Sanipour F, Moss T (2014). Existence of benefit finding and posttraumatic growth in people treated for head and neck cancer: A systematic review. PeerJ.

[63] Liu Z, Thong MS, Doege D (2023). Benefit finding, posttraumatic growth and health-related quality of life in long-term cancer survivors: A prospective population-based study. Acta Oncol.

[64] Ho SM, Chan CL, Ho RT (2004). Posttraumatic growth in Chinese cancer survivors. Psychooncology.

[65] Taku K, Calhoun LG, Tedeschi RG, Gil-Rivas V, Kilmer RP, Cann A (2007). Examining posttraumatic growth among Japanese university students. Anxiety Stress Coping.

[66] Exenberger S, Kumnig M, Juen B, Rumpold G, Siller H (2019). Dimensions of posttraumatic growth in a German-speaking sample using mixed methods. Eur J Psychotraumatol.

